# Characterization of Different Subtypes of Immune Cell Infiltration in Glioblastoma to Aid Immunotherapy

**DOI:** 10.3389/fimmu.2022.799509

**Published:** 2022-06-21

**Authors:** Peng Feng, Zhenqing Li, Yuchen Li, Yuelin Zhang, Xingyu Miao

**Affiliations:** ^1^ Neurosurgery Shaanxi Provincial People’s Hospital, Xi’an, China; ^2^ Medical College of Nantong University, Nantong, China; ^3^ Hengyang Medical School, University of South China, Hengyang, China; ^4^ Graduate Office Xi’an Medical University, Xi’an, China

**Keywords:** GBM, PD-1 inhibitor, immune cell infiltration, TCGA, immunotherapy

## Abstract

Glioblastoma multiforme (GBM) has been identified as a frequently occurring adult primary brain cancer that is highly aggressive. Currently, the prognostic outcome for GBM patients is dismal, even with intensive treatment, and the median overall survival (OS) is 14.6 months. Immunotherapy, which is specific at the cellular level and can generate persistent immunosurveillance, is now becoming a promising tool to treat diverse cancers. However, the complicated nature of the tumor microenvironment (TME) makes it challenging to develop anti-GBM immunotherapy because several cell types, cytokines, and signaling pathways are involved in generating the immunosuppressive environment. Novel immunotherapies can illustrate novel tumor-induced immunosuppressive mechanisms. Here, we used unsupervised clustering analysis to identify different subtypes of immune cell infiltration that actuated different prognoses, biological actions, and immunotherapy responses. Gene cluster A, with a hot immune cell infiltration phenotype, had high levels of immune-related genes (IRGs), which were associated with immune pathways including the interferon-gamma response and interferon-alpha response, and had low *IDH1* and *ATRX* mutation frequencies. Gene cluster B, a cold immune cell infiltration subtype, exhibited a high expression of the *KCNIP2*, *SCRT1*, *CPLX2*, *JPH3*, *UNC13A*, *GABRB3*, *ARPP21*, *DLGAP1*, *NRXN1*, *DLL3*, *CA10*, *MAP2*, *SEZ6L*, *GRIA2*, and *GRIA4* genes and a low expression of immune-related genes, i.e., low levels of immune reactivity. Our study highlighted the complex interplay between immune cell infiltration and genetic mutation in the establishment of the tumor immune phenotype. Gene cluster A was identified as an important subtype with a better prognosis and improved immunotherapy response.

## Introduction

Glioblastoma multiforme (GBM) is the most aggressive and most lethal primary brain tumor and often occurs as a primary brain cancer in adults (1). GBM tumors are characterized by aggressive growth, intensive angiogenesis, and poor prognosis. The median survival is approximately 15 months when a modern protocol of complex treatment is used (1–3). Currently, there is no efficient therapy available for relapsed or advanced GBM cases. Due to the plasticity and proliferation capacity of tumor stem cells, GBM has strong radiation resistance, and radiotherapy and chemotherapy cannot effectively eliminate CSCs (4). Thus, the development of fundamentally novel approaches to GBM treatment is needed. Immunotherapy (IT) is one such treatment with great potential. Over the last decade, immune checkpoint inhibitor (ICI)-based immunotherapies have achieved immense success in the treatment of diverse cancers. Nonetheless, according to a recent clinical trial, an objective response was achieved in just 8% of cases in which the ICI programmed cell death 1 (PD-1) was used to treat relapsed GBM. A recent study highlighted the importance of GBM heterogeneity, which makes this kind of cancer among the most challenging to treat (5). The different patterns of heterogeneity in glioma show different chromosome, genome, and transcriptome characteristics (5) and different patterns of biomarkers and driver genes (6). Based on these characteristics, we can obtain different effective and detailed targets for different biological models of GBM. Emerging evidence suggests that intratumoral heterogeneity may also influence tumor immune responses. Cancer heterogeneity may influence antigens and particular gene mutations, which can be recognized by the immune system and then regulate the immune response (7). However, more efforts are needed to explain the underlying mechanism associated with alterations in response patterns. The improved responses to anti-PD-1 therapy have been related to greater tumor mutation burdens among diverse cancers as well as T-cell infiltrating degrees within the tumor microenvironment (TME). Defining reliable targets for the prediction of the ICI response is still a major challenge because of the dynamic nature and complexity of the immune response in the TME (8, 9). Several studies have shown that primary GBM can be divided into distinct immune subgroups, which can be defined by gene expression profiles (10, 11). However, the underlying molecular principles driving the establishment and maintenance of the tumor immune phenotype are still not addressed in detail. Here, we aim to reveal the complexity of immune cell infiltration and epigenetic alterations and their impact on the establishment and maintenance of the tumor immune microenvironment and tumor prognosis. Our study not only showed the molecular mechanism of how cancer cells evade immune surveillance but also identified the key genes that regulate immune cell infiltration and activate immune-related pathways. Many studies have shown that metastasis is the most lethal development, especially brain metastasis. Many works have reported that lung adenocarcinoma, breast cancer, and bladder cancer can produce brain metastasis at a significant frequency (12–15) and cause poor treatment outcomes and poor prognosis (12, 14, 16). Esophageal cancer can affect intestinal microorganisms (17, 18) and indirectly promote the development of GBM, leading to a poor prognosis (19–25). Low-grade glioma can also cause secondary GBM (26). Thus, we conducted an external validation [in esophageal carcinoma (ESCA), low-grade glioma (LGG), lung adenocarcinoma (LUAD), breast cancer (BRCA), and bladder cancer (BLAD) cohorts] to verify the ability of those gene clusters to predict the prognosis of other tumors. Our findings showed that the activated key genes have the potential to improve the risk assessment of GBM under immunotherapy and enable early stratification of patients with cancer at a higher risk for treatment failure who may benefit from combination therapy targeting newly identified key genes.

## Materials and Methods

### Datasets and Data Normalization

The gene expression matrix of 1,092 patients and complete survival information were downloaded as follows: 145 samples (TCGA-GBM) from The Cancer Genome Atlas (TCGA) database (https://portal.gdc.cancer.gov/), 693 samples from the Chinese Glioma Genome Atlas (CGGA)-GBM, and 254 samples from the Gene Expression Omnibus (GEO) datasets (GSE7696 and GSE4412). Detailed survival data were available for 474 samples, including 141 samples in TCGA, 195 samples in CGGA, and 137 samples in the GEO datasets. Normalization of the data was conducted after multiple datasets were merged, and then the expression values were log-transformed. We used the “ComBat” algorithm to reduce the impact of the probable batch effects (27). Furthermore, we obtained segment data on copy numbers at FireBrowse (http://firebrowse.org), and the immune cell marker genes were acquired from the TISIDB (http://cis.hku.hk/TISIDB/data/download/CellReports.txt). The gene expression matrix and related clinical data of TCGA-BRCA, TCGA-LUAD, TCGA-BLAD, TCGA-ESCA, and TCGA-LGG were acquired from the TCGA database.

### Consensus Clustering for Different Immune Cell Infiltration Subtypes

We used consensus clustering to stratify and cluster those samples based on the immune cell infiltration pattern with a specific pattern in which the “km” analysis, Pearson’s distance, and Ward’s linkage-based unsupervised clustering approach were conducted by the “Consensus Cluster Plus” R package (28). To ensure clustering stability, we repeated the aforementioned process approximately 100 times.

### Differentially Expressed Genes Associated With the Immune Cell Infiltration Phenotype

We used the limma package (29) to acquire the differentially expressed genes (DEGs) between the two clusters (cluster A and cluster B). The absolute fold-change cutoff was designated as >1, and *P <*0.05 was identified as significant.

### Consensus Clustering for Different Gene Subtypes

In order to reveal the correlation between the tumor immune subtypes and epigenetic events, we used consensus clustering with “PAM” analysis (parameters: reps, 100; pItem, 0.8; pFeature, 1; Ward.D2 and Euclidean distance, *k* = 3) based on those DEG expression matrix data to stratify those samples into different gene subtypes. The aforementioned process was repeated 100 times to ensure clustering stability.

### Gene Set Variation Analysis and Functional Annotation

To explore the biological event differences in different subtypes, the gene set variation analysis (GSVA) package in R (30) software was used to conduct the biological function analysis (11). In addition, “h.all.v7.5.1.symbols.gmt” was downloaded from the MSigDB dataset, and *P <*0.5 was considered significant.

### Collection of Somatic Alteration Data

The mutation data were acquired from TCGA-GBM (https://portal.gdc.cancer.gov/), and we evaluated the total number of non-synonymous mutations in GBM. Then, we used the “maftools” R package (https://rdrr.io/bioc/maftools/) to identify the significantly mutated genes between different gene clusters based on the top 80% of the genes with the highest alteration frequency. A *P*-value <0.05 was identified as statistically significant.

### Chemotherapeutic Response Prediction

Based on the largest public pharmacogenomics database, namely, the Genomics of Drug Sensitivity in Cancer (GDSC), the “pRRophetic” function of the R package (31) was adopted for predicting chemotherapeutic responses to paclitaxel and cisplatin in every sample. The package determined the half-maximal inhibitory concentration (IC_50_) of GDSC training set samples through 10-fold cross-validation (13).

### Copy Number Variation Analysis

Copy number variation (CNV) data (GBM.snp:genome_wide_snp_6:broad_mit_edu:Level_3:segmented_scna_minus_germline_cnv_hg18:seg.seg) were acquired from http://www.firebrowse.org/. A segment_Mean >0.1 was identified as a gain and a value less than −0.1 as a loss. We used the package “BSgenome.Hsapiens.UCSC.hg19” to analyze the copy number variation of different subtypes.

### Gene Expression Data After Immunotherapy

For further investigation of immunotherapy response, we downloaded the IMvigor210 datasets, which include data from 298 urothelial cancer patients who received immunotherapy and detailed information about the response to PD-L1 blockade; these data can be obtained from an accessible, well-documented software and data package (32). Then, we used the IMvigor210 datasets to analyze the value of different gene clusters in the predicted PD-1 response.

### Statistical Analysis

The R 4.0.0 package was used for all statistical analyses. The Wilcoxon signed-rank test was carried out for pairwise comparative analysis, whereas multiple groups were compared using the Kruskal–Wallis test (33). The false discovery rates (FDRs) in limma and gene set enrichment analysis (GSEA) were adjusted by the Benjamini–Hochberg approach at the *P <*0.05 significance level. The “survival” package (34) was used to determine the difference in survival between different subtypes. The correlation between categorical clinical information and the defined clusters was statistically examined by Fisher’s exact test. *P <*0.05 was identified as statistically significant.

## Results

### Different Immune Cell Infiltration Profiles

**Figure 1:**
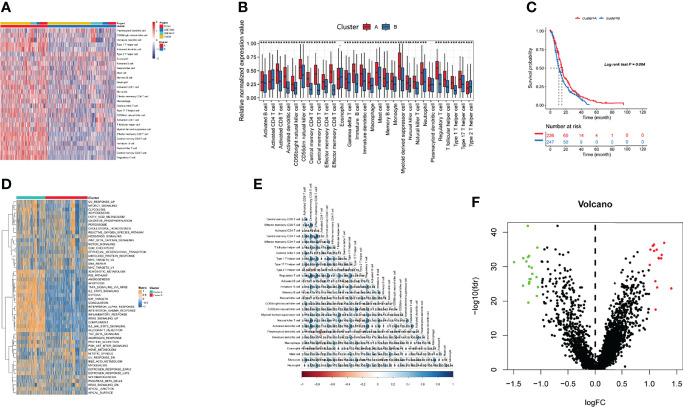
Identification of immunogenic subtypes. **(A)** Landscapes for the 28 infiltrating immune cell types of both clusters. **(B)** The distribution of tumor-infiltrating immune cells in the two clusters. **(C)** Survival analysis of the two clusters. **(D)** Heatmaps of 50 hallmark pathways with differential activation in different clusters. **(E)** The correlations with different immune cells. **(F)** Differentially expressed genes in the two clusters. (***p ≤ 0.001).

Four gene expression datasets, including the TCGA-GBM, CGGA-GBM, and GEO datasets (GSE7696 and GSE4412), comprising 1,092 samples in total, were subjected to ssGSEA computational algorithms to predict the immune cell infiltration score. Based on the 28 immune cell types, we used unsupervised hierarchical cluster analysis to identify two main clusters: A and B (**Figure 1A**). Cluster A exhibited high infiltration by the activation of 28 immune cells (**Figure 1B**). Cluster A was also associated with a better survival rate (**Figure 1C**) and was enriched in the interferon-alpha response, interferon-gamma response, and IL2 stat5 signaling (**Figure 1D**), which demonstrated that cluster A more readily produced an immune response than cluster B. Furthermore, this study derived a correlation coefficient heatmap for visualizing 28 tumor-infiltrating immune cell types (**Figure 1E**). The two clusters had 34 DEGs (differentially expressed genes) (**Table S1**), as shown in **Figure 1F**.

### Identified Gene Subtypes Based on the DEGs

**Figure 2:**
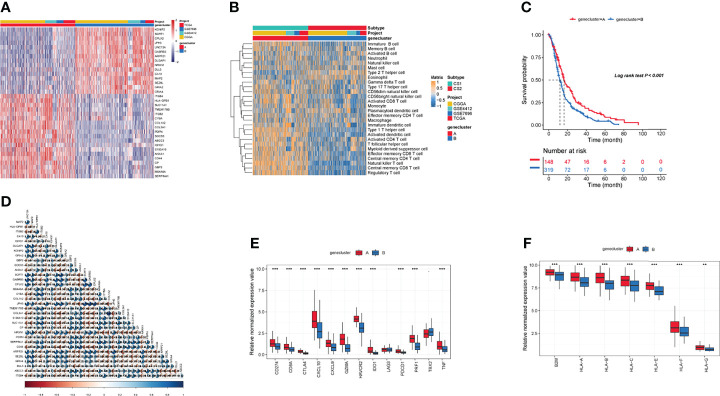
Identification of gene subtypes. **(A)** The landscapes of 34 differentially expressed genes in the two gene clusters. **(B)** Heatmaps of infiltration of 28 immune cell types in different gene clusters. **(C)** Survival analysis in the two gene clusters. **(D)** The correlation in 34 genes in the tumor patients. **(E)** Expression of genes related to immune activation (*CD8A*, *CXCL9*, *CXCL10*, *GZMA*, *GZMB*, *IFNG*, *PRF1*, *TNF*, *TBX2*) and immune checkpoints (*CD274*, *IDO1*, *PDCD1*, *HAVCR2*, *LAG3*, *CTLA4*) within the two gene clusters. **(F)** The distribution of *B2M* and *HLA* genes in the two gene clusters. (**p ≤ 0.01; ***p ≤ 0.001).

**Figure 3:**
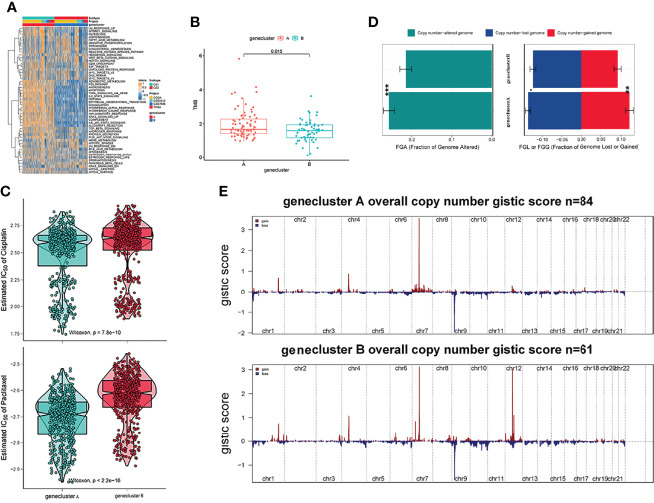
Genetic alteration landscape among glioblastoma multiforme (GBM) genomic subtypes. **(A)** Heatmaps of 50 hallmark pathways with differential activation in different gene clusters. **(B)** The distribution of tumor burden mutations in different gene clusters. **(C)** Patients with gene cluster A are likely to receive greater benefit from cisplatin and paclitaxel treatment. **(D)** Fraction genome gain/loss (FGA/FGG) and fraction genome altered (FGA) distributions. Bar charts show the mean ± SEM. **(E)** The G score plot of the deleted or amplified genomic regions in gene clusters was determined using GISTIC 2.0. The G score was determined by multiplying copy numbers by the frequency across cases. (**p ≤ 0.01; ***p ≤ 0.001).

Recent studies have used gene expression data to define different immune subtypes and divide tumor patients into immunotherapy response groups (10, 11). Thus, we used consensus clustering analysis of the gene expression data of 34 DEGs to identify different gene clusters (gene cluster A and gene cluster B) (**Figure 2A**). Gene cluster A can be defined as a hot immune cell infiltration type (**Figure 2B**), associated with a better survival rate (**Figure 2C**). Moreover, we pictured a correlation coefficient heatmap for visualizing 34 gene expression levels (**Figure 2D**). Furthermore, immune-related genes, including *CD274*, *CTLA4*, *HAVCR2*, *IDO1*, *LAG3*, and *PDCD1*, were identified as immune checkpoint-relevant signature genes, and *CD8A*, *CXCL10*, *CXCL9*, *GZMA*, *GZMB*, *IFNG*, *PRF1*, *TBX2*, and *TNF* (immune-activity-relevant signature genes) were overexpressed in gene cluster A compared with the other subtypes. This indicated that gene cluster A is associated with high levels of immune reactivity (**Figure 2E**). Because MHC class I complexes present tumor-associated antigens, they are essential for immune surveillance and can help to improve the clinical responses of immunotherapy targeting immune checkpoints (35). Here, we assessed the *B2M* and *HLA* gene expression levels in the two gene clusters. The results showed that the expression of all *B2M* and *HLA* genes was progressively reduced in gene cluster B, suggesting that impaired antigen presentation may be the evasion mechanism of gene cluster B in the immune response (**Figure 2F**). First, we compared the activation status of 50 tumorigenesis-related pathways in the two gene clusters. We observed the significance of the IL6–JAK–STAT3 signaling, interferon-alpha response, and interferon-gamma response pathways in gene cluster B (**Figure 3A**). *IFNG* can regulate innate and adaptive immune responses (35–37), and increasing evidence has identified that *IFNG* can enhance antigen presentation, T-lymphocyte differentiation, and maturation to activate the tumor immune response (36, 38, 39). In our study, gene cluster A had abundant immune cell infiltration and strong immune pathway activity, which indicated that gene cluster A is a better fit for immunotherapy. Genetic alterations significantly affect the formation of tumor genetic subtypes. As **Figure 3B** shows, we found that gene cluster B had the highest total tumor mutation load. Gene cluster B had high mutation frequencies of *IDH1* (12.1%) and *ATRX* (12.1%) (**Table 1**), indicating that patients in gene cluster B with ICI treatment failure may benefit from combined inhibition with *IDH1* and *ATRX*. Furthermore, we evaluated the IC_50_ of paclitaxel and cisplatin, and the study indicated that the gene cluster A patients showed stronger responses (*P* < 0.05) to paclitaxel and cisplatin therapy than did the gene cluster B patients (**Figure 3C**). We also assessed the copy number changes in the two subtypes, and significant increases in both copy loss and gain were found for gene cluster A compared to gene cluster B (**Figure 3D**). For gene cluster B, copy number variation was primarily observed within the 12q13.2 chromosomal region, while no such variation in this region was observed for gene cluster A (**Figure 3E**).

**Table 1:** Association of different subtypes with somatic variants.

Gene	Gene cluster A	Gene cluster B	*P*-value
*TPTE2* (transmembrane phosphoinositide 3-phosphatase and tensin homolog 2)	3 (3.8%)	4 (6.9%)	4.56E−01
*SLCO6A1* (solute carrier organic anion transporter family member 6A1)	3 (3.8%)	4 (6.9%)	4.56E−01
*COL6A2* (collagen type VI alpha 2 chain)	6 (7.6%)	2 (3.4%)	4.67E−01
*PIK3CA* (phosphatidylinositol-4,5-bisphosphate 3-kinase catalytic subunit alpha)	4 (5.1%)	7 (12.1%)	2.03E−01
*ATRX* (ATRX chromatin remodeler)	2 (2.5%)	7 (12.1%)	3.61E−02
*SEMA3C* (semaphorin 3C)	3 (3.8%)	4 (6.9%)	4.56E−01
*DCHS2* (dachsous cadherin-related 2)	6 (7.6%)	2 (3.4%)	4.67E−01
*IDH1* (isocitrate dehydrogenase (NADP(+)) 1)	0 (0.0%)	7 (12.1%)	1.95E−03
*OBSCN* (obscurin, cytoskeletal calmodulin and titin-interacting RhoGEF)	8 (10.1%)	3 (5.2%)	3.55E−01
*TP53* (tumor protein p53)	24 (30.4%)	23 (39.7%)	2.79E−01
*TAF1L* (TATA-box binding protein associated factor 1 like)	2 (2.5%)	5 (8.6%)	1.33E−01
*AHNAK2* (AHNAK nucleoprotein 2)	7 (8.9%)	8 (13.8%)	4.13E−01
*PCDHA10* (protocadherin alpha 10)	3 (3.8%)	4 (6.9%)	4.56E−01
*LAMA1* (laminin subunit alpha 1)	4 (5.1%)	5 (8.6%)	4.94E−01
*MYH2* (myosin heavy chain 2)	6 (7.6%)	2 (3.4%)	4.67E−01
*DNAH8* (dynein axonemal heavy chain 8)	3 (3.8%)	4 (6.9%)	4.56E−01
*VWF* (von Willebrand factor)	2 (2.5%)	5 (8.6%)	1.33E−01
*PCLO* (piccolo presynaptic cytomatrix protein)	3 (3.8%)	9 (15.5%)	2.87E−02
*ARHGEF5* (Rho guanine nucleotide exchange factor 5)	3 (3.8%)	4 (6.9%)	4.56E−01
*NF1* (neurofibromin 1)	10 (12.7%)	3 (5.2%)	2.37E−01
*RB1* (RB transcriptional corepressor 1)	9 (11.4%)	3 (5.2%)	2.38E−01
*PCDHA12* (protocadherin alpha 12)	3 (3.8%)	4 (6.9%)	4.56E−01
*APOB* (apolipoprotein B)	6 (7.6%)	2 (3.4%)	4.67E−01
*SPTA1* (spectrin alpha, erythrocytic 1)	6 (7.6%)	8 (13.8%)	2.65E−01
*FAT2* (FAT atypical cadherin 2)	7 (8.9%)	2 (3.4%)	3.01E−01

### Immunotherapy Response

**Figure 4:**
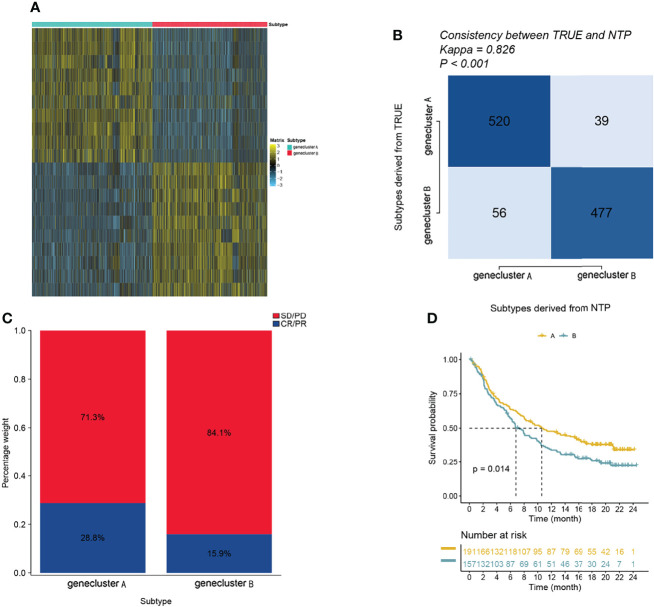
The role of gene clusters in the prediction of immunotherapeutic benefits. **(A)** Heatmap showing significantly upregulated biomarkers with subtype specificity examined by limma for GBM subtypes. **(B)** Consistency heatmap using Kappa statistics. **(C)** Different subtypes with varying anti-PD-1 responses. **(D)** Kaplan–Meier graphs of different subtypes in the IMvigor210 cohort.

Since transcriptomic data are extensively utilized in cancer studies, we evaluated 100 specific genes with significant upregulation as classifiers for all subtypes based on the GBM cohort. We produced genetic signatures composed of these sets of 100 genes to predict the subtypes of GBM cases in the external datasets (**Figures 4A, B**). The results revealed that the cluster A subtype had a high CR/PR of 28.3% compared with the cluster B subtype (**Figure 4C**). In the IMvigor210 cohort, the cluster A subtype had an increased OS rate compared with additional subtypes from anti-PD-1 treatment (**Figure 4D**).

### Validation of Diverse Clinical Outcomes of Subtypes in External Cohorts

**Figure 5:**
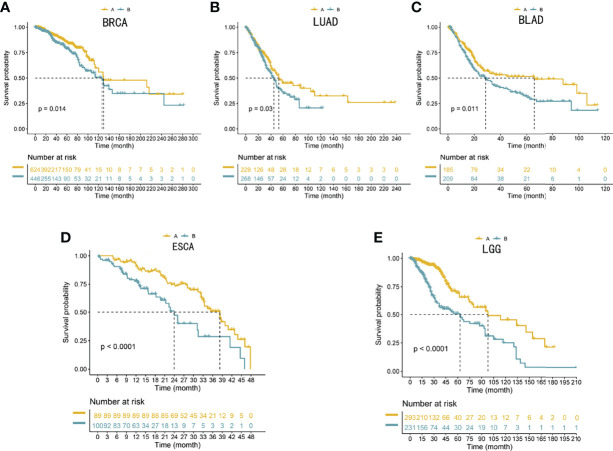
**(A–E)** Validation of the 100-gene signature to reproduce the five gene subtypes in external cohorts. Log-rank tests and Kaplan–Meier curves were adopted for displaying and comparing the OS between the two subtypes. The Benjamini–Hochberg step-up method was utilized to adjust the *P*-values in the two subtypes.

To reproduce those two subtypes, the 100 most significant genes in every subtype relative to the remaining two were selected and assigned as gene clusters A and B. Then, the clinical outcomes of cases in the BRCA, LUAD, BLAD, ESCA, and LGG cohorts were compared. Patients showed similar survival rates regardless of cluster assignment, although those from cluster B showed the poorest clinical outcomes (*P* < 0.05, **Figure 5**).

## Discussion

### Glioblastoma

GBM is a common intracranial tumor with a high degree of malignancy; it is characterized by fast growth, strong invasion, a high frequency of recurrence, and poor prognosis (40, 41). The treatment of GBM poses two main problems. First, because of the high malignancy, rapid progression, 3–5-day cancer cell cycle, and short disease course, GBM relapse after even radical surgery becomes inevitable. Second, cancer cells adapt to potentially invade the surrounding non-carcinoma tissues; they can approach some lobes, deeper structures, or the contralateral hemisphere *via* the corpus callosum (42, 43). Hence, it is imperative to avoid metastasis and improve prognosis in patients with GBM. Previous clinical trials of immunotherapies suggest that immunotherapy is highly effective in inhibiting cancer progression and improving patient quality of life. Recently, significant advances have been made in immune cell infiltration into central nervous system (CNS) tumors, but immunotherapy still shows a low response rate in GBM. Thus, it is urgent to understand the molecular mechanisms underlying immune cell infiltration and immune escape and the related gene expression characteristics of the tumor cells themselves and the TME to improve the effect of ICI therapy. Here, we used unsupervised hierarchical cluster analysis to identify two different immune cell infiltration clusters. Variations in prognosis, immune cell infiltration, and underlying biological function among distinct subgroups were assessed. Cluster A, also called the inflammatory subtype, had high expression levels of immune-related genes; high activation of immune-associated pathways such as the interferon-alpha response, interferon-gamma response, and IL2 stat5 signaling; and a better survival rate. Conversely, cluster B demonstrated the worst prognosis, which may be impacted by tumor-related pathways such as apoptosis, hypoxia, and E2F targets.

Thirty-four DEGs between two immune subtypes showed clear differential expression between hot immune cell infiltration (cluster A) and cold immune cell infiltration (cluster B). For establishing the relationship between tumor immune subtypes and epigenetic events, we identified the different gene clusters. Gene cluster A can be identified as a hot immune subtype, characterized by better prognosis, high expression levels of immune-related genes, and low mutation frequencies of *IDH1* and *ATRX.* A recent study showed that *IDH1* and *ATRX* mutations can regulate the innate immune response in gliomas, which also enhances the invasiveness of gliomas and facilitates glioma development (44, 45). Gene cluster B had high mutation frequencies of *IDH1* (12.1%) and *ATRX* (12.1%) (**Table 1**), which indicated that patients in gene cluster B with ICI treatment failure may benefit from combined treatment with *IDH1* and *ATRX* inhibitors. Gene cluster B can be called the cold immune subtype, associated with poor prognosis, and we found that the 12q13.2 region gain is associated with gene cluster B. Chemotherapy remains the principal method for cancer treatment, with paclitaxel and cisplatin being the classical drugs. We also observed that the gene cluster A patients were more likely to respond to paclitaxel and cisplatin therapy (*P* < 0.05) than the gene cluster B patients. Among the two gene clusters, the immune-related genes (*CD274*, *CTLA4*, *HAVCR2*, *IDO1*, *LAG3*, and *PDCD1*) and immune activity-relevant genes (*CD8A*, *CXCL10*, *CXCL9*, *GZMA*, *GZMB*, *IFNG*, *PRF1*, *TBX2*, and *TNF*) were expressed at higher levels in the gene cluster A subtype than in the other subtypes, indicating that gene cluster A has a high level of immunoreactivity, which means that it has a high level of immune activation and can then elicit an effective immune response. The evaluation of patients receiving immunotherapy by IMvigor210 showed a remarkable enrichment of gene cluster A in patients who responded to immunotherapy (28.3%), confirming the predictive value of this genetic profile. This suggests that the existing immunity has anticancer activity, which may have a positive effect on the immunotherapy response.

Many studies have reported that lung adenocarcinoma, breast cancer, and bladder cancer can significantly produce brain metastasis (13–15) and cause poor treatment outcomes and poor prognosis (12, 14, 16). A recent study showed that the gut microbiota can promote the development of glioma (22, 24), especially with respect to cell proliferation and tumor invasiveness phenotypes (19–21). The gut microbiota presents unique changes in esophageal cancer and can indirectly cause the deterioration of GBM and lead to a poor prognosis of GBM (19–25). Recent studies have shown that low-grade glioma can also cause secondary GBM (26). To evaluate the ability of those gene clusters to predict the prognosis of other tumors, we conducted an external validation (ESCA, LGG, LUAD, BRCA, and BLAD cohorts). We selected the top 100 specific genes with the highest values in each subtype to reproduce the two gene clusters, representing the separation of gene cluster A and gene cluster B. The clinical prognosis of gene cluster A was better in all five external cohorts (*P* < 0.05, **Figure 5**).

In our study, gene cluster A could be classified as an effective immune response subtype, demonstrating that GBM samples with a gene cluster A-related gene expression signature have effective cytotoxic immune cells. Through a comprehensive analysis of multiple omics data, we revealed the high mutation frequency of *IDH1* and *ATRX* mutations as a marker of gene cluster B, taking into account genetic and epigenetic changes as well as the effects of gene mutations. This observation indicates that patients with GBM at a higher risk for treatment failure might benefit from targeted *IDH1* and *ATRX* inhibition in combination with ICI. Our study differs from other recently published studies (11) mainly because we performed a comprehensive analysis of multiple omics data to highlight the association between tumor immune subtypes and epigenetic patterns. We identified gene cluster A as a key tumor immune response subtype, with high levels of tumor-infiltrating cytotoxic immune cells and consequent high efficacy of ICI therapy.

While the existing studies mainly focus on the expression of specific genes (46), our study mainly focused on subtypes that were constructed by the expression profiles of multiple genes and revealed the various mechanisms of immune regulation and the tumor immune response. This system may be more stable and less sensitive to changes in the expression of single genes.

**Figure 6:**
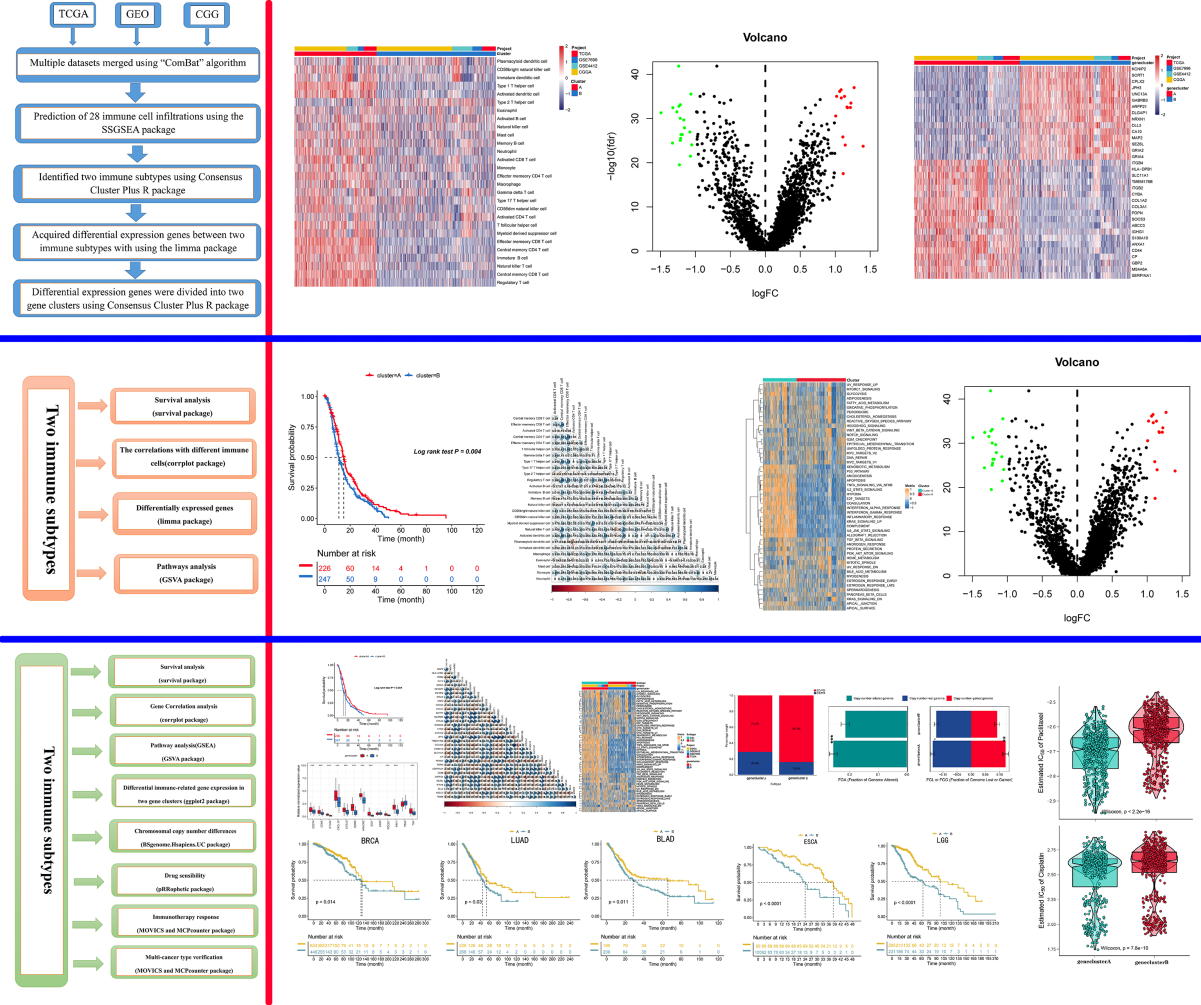
Graphical abstract. The model of a complex multi-omics regulation of the tumor immune phenotype in GBM.

In summary, this study comprehensively analyzes the immune cell infiltration pattern in GBM and sheds more light on pro-/antitumor immune modulation within GBM. Differences in gene expression patterns were associated with tumor heterogeneity and treatment complexity. Hence, this study identified a key gene expression subtype that can be considered relevant to immunotherapy and chemotherapy response and can help promote the individualized treatment of tumors (**Figure 6**).

## Data Availability Statement

The datasets presented in this study can be found in online repositories. The names of the repository/repositories and accession number(s) can be found in the article/**Supplementary Material**.

## Author Contributions

PF and XM conceived and designed the study, obtained funding, and drafted the manuscript. PF and ZL acquired the data and drafted the manuscript. PF critically revised the manuscript. ZL,YL and YZ performed statistical analysis and technical support. All authors contributed to the article and approved the submitted version.

## Funding

This study was supported by a grant (grant number: 2022SF-166) from the Key Project of Shaanxi Province-the field of Social Development.

## Conflict of Interest

The authors declare that the research was conducted in the absence of any commercial or financial relationships that could be construed as a potential conflict of interest.

## Publisher’s Note

All claims expressed in this article are solely those of the authors and do not necessarily represent those of their affiliated organizations, or those of the publisher, the editors and the reviewers. Any product that may be evaluated in this article, or claim that may be made by its manufacturer, is not guaranteed or endorsed by the publisher.
