# Supplementary material for: Characterization of Different Subtypes of Immune Cell Infiltration in Glioblastoma to Aid Immunotherapy
Source: Front Immunol. 2022 Jun 21;13:799509. doi: 10.3389/fimmu.2022.799509 (PMC9254719; doi:10.3389/fimmu.2022.799509)
Supplement: Supplementary file 1 [file Table_1.docx]

Table S1: Differential expression genes in different gene clusters.

| **gene** | **conMean** | **treatMean** | **logFC** | **pValue** | **fdr** |
| --- | --- | --- | --- | --- | --- |
| CPLX2 ([complexin 2](https://www.ncbi.nlm.nih.gov/gene/10814)) | 4.424968973 | 5.824318603 | 1.39934963 | 6.03E-26 | 1.92E-24 |
| SEZ6L ([seizure related 6 homolog like](https://www.ncbi.nlm.nih.gov/gene/23544)) | 4.787541996 | 6.057908075 | 1.270366079 | 7.92E-41 | 1.03E-37 |
| JPH3 ([junctophilin 3](https://www.ncbi.nlm.nih.gov/gene/57338)) | 3.88598574 | 5.110396719 | 1.224410979 | 1.36E-36 | 3.32E-34 |
| SCRT1 ([scratch family transcriptional repressor 1](https://www.ncbi.nlm.nih.gov/gene/83482)) | 2.874303897 | 4.091425895 | 1.217121999 | 1.86E-35 | 3.32E-33 |
| MAP2 ([microtubule associated protein 2](https://www.ncbi.nlm.nih.gov/gene/4133)) | 6.505247148 | 7.680325692 | 1.175078544 | 2.16E-35 | 3.58E-33 |
| KCNIP2 ([potassium voltage-gated channel interacting protein 2](https://www.ncbi.nlm.nih.gov/gene/30819)) | 4.330403214 | 5.499060837 | 1.168657623 | 1.39E-35 | 2.59E-33 |
| CA10 ([carbonic anhydrase 10](https://www.ncbi.nlm.nih.gov/gene/56934)) | 3.551472324 | 4.694207596 | 1.142735271 | 2.97E-26 | 1.03E-24 |
| GABRB3 ([gamma-aminobutyric acid type A receptor subunit beta3](https://www.ncbi.nlm.nih.gov/gene/2562)) | 3.333981995 | 4.466875963 | 1.132893968 | 2.71E-38 | 1.00E-35 |
| DLL3 ([delta like canonical Notch ligand 3](https://www.ncbi.nlm.nih.gov/gene/10683)) | 5.987860249 | 7.101962802 | 1.114102553 | 2.08E-19 | 2.96E-18 |
| GRIA2 ([glutamate ionotropic receptor AMPA type subunit 2](https://www.ncbi.nlm.nih.gov/gene/2891)) | 5.3231974 | 6.432265835 | 1.109068436 | 2.76E-28 | 1.52E-26 |
| ARPP21 ([cAMP regulated phosphoprotein 21](https://www.ncbi.nlm.nih.gov/gene/10777)) | 3.877249307 | 4.964504243 | 1.087254935 | 6.96E-38 | 2.27E-35 |
| UNC13A ([unc-13 homolog A](https://www.ncbi.nlm.nih.gov/gene/23025)) | 3.663106241 | 4.746810926 | 1.083704685 | 3.27E-40 | 3.20E-37 |
| NRXN1 ([neurexin 1](https://www.ncbi.nlm.nih.gov/gene/9378)) | 4.25641192 | 5.320034479 | 1.063622559 | 9.59E-40 | 7.51E-37 |
| DLGAP1 ([DLG associated protein 1](https://www.ncbi.nlm.nih.gov/gene/9229)) | 3.846994364 | 4.867119811 | 1.020125447 | 2.24E-38 | 9.76E-36 |
| GRIA4 ([glutamate ionotropic receptor AMPA type subunit 4](https://www.ncbi.nlm.nih.gov/gene/2893)) | 4.310489896 | 5.317224907 | 1.006735011 | 2.24E-33 | 2.74E-31 |
| ITGB4 ([integrin subunit beta 4](https://www.ncbi.nlm.nih.gov/gene/3691)) | 5.836261148 | 4.784218313 | -1.052042835 | 2.55E-26 | 9.06E-25 |
| SERPINA1 ([serpin family A member 1](https://www.ncbi.nlm.nih.gov/gene/5265)) | 5.14436971 | 4.079794346 | -1.064575364 | 4.77E-39 | 2.95E-36 |
| S100A10 ([S100 calcium binding protein A10](https://www.ncbi.nlm.nih.gov/gene/6281)) | 7.876609515 | 6.8050741 | -1.071535416 | 1.40E-23 | 3.41E-22 |
| CYBA ([cytochrome b-245 alpha chain](https://www.ncbi.nlm.nih.gov/gene/1535)) | 8.086932829 | 7.007533196 | -1.079399633 | 1.59E-29 | 1.13E-27 |
| CD44 ([CD44 molecule (Indian blood group)](https://www.ncbi.nlm.nih.gov/gene/960)) | 6.36362868 | 5.206553395 | -1.157075285 | 1.64E-30 | 1.31E-28 |
| GBP2 ([guanylate binding protein 2](https://www.ncbi.nlm.nih.gov/gene/2634)) | 5.5952697 | 4.428430577 | -1.166839124 | 1.87E-32 | 2.09E-30 |
| PDPN ([podoplanin](https://www.ncbi.nlm.nih.gov/gene/10630)) | 5.258381936 | 4.070403202 | -1.187978734 | 7.16E-29 | 4.28E-27 |
| SOCS3 (suppressor of cytokine signaling 3) | 5.06042846 | 3.854095438 | -1.206333022 | 7.22E-29 | 4.28E-27 |
| HLA-DPB1 ([major histocompatibility complex, class II, DP beta 1](https://www.ncbi.nlm.nih.gov/gene/3115)) | 6.652324271 | 5.441487484 | -1.210836786 | 6.21E-28 | 3.15E-26 |
| ITGB2 ([integrin subunit beta 2](https://www.ncbi.nlm.nih.gov/gene/3689)) | 6.489642776 | 5.273381891 | -1.216260885 | 3.50E-36 | 7.60E-34 |
| COL1A2 ([collagen type I alpha 2 chain](https://www.ncbi.nlm.nih.gov/gene/1278)) | 5.935116609 | 4.718417433 | -1.216699176 | 2.03E-27 | 9.34E-26 |
| TMEM176B ([transmembrane protein 176B](https://www.ncbi.nlm.nih.gov/gene/28959)) | 6.572042344 | 5.35470235 | -1.217339994 | 7.94E-33 | 9.14E-31 |
| ANXA1 ([annexin A1](https://www.ncbi.nlm.nih.gov/gene/301)) | 6.412894135 | 5.183859428 | -1.229034707 | 1.59E-21 | 3.00E-20 |
| MS4A6A (membrane spanning 4-domains A6A) | 6.373048538 | 5.13778264 | -1.235265897 | 8.43E-36 | 1.65E-33 |
| SLC11A1 ([solute carrier family 11 member 1](https://www.ncbi.nlm.nih.gov/gene/6556)) | 4.886544039 | 3.646713063 | -1.239830976 | 7.24E-46 | 1.42E-42 |
| CP ([ceruloplasmin](https://www.ncbi.nlm.nih.gov/gene/1356)) | 4.694777277 | 3.429498106 | -1.265279171 | 2.04E-34 | 3.06E-32 |
| ABCC3 ([ATP binding cassette subfamily C member 3](https://www.ncbi.nlm.nih.gov/gene/8714)) | 4.193261453 | 2.869980713 | -1.32328074 | 2.19E-35 | 3.58E-33 |
| COL3A1 ([collagen type III alpha 1 chain](https://www.ncbi.nlm.nih.gov/gene/1281)) | 5.118399214 | 3.790217951 | -1.328181263 | 8.83E-27 | 3.64E-25 |
| IGHG1 ([immunoglobulin heavy constant gamma 1 (G1m marker)](https://www.ncbi.nlm.nih.gov/gene/3500)) | 4.120355812 | 2.628717029 | -1.491638783 | 3.97E-34 | 5.18E-32 |
